# Fears and Misperceptions of the Ebola Response System during the 2014-2015 Outbreak in Sierra Leone

**DOI:** 10.1371/journal.pntd.0005077

**Published:** 2016-10-18

**Authors:** Thespina Yamanis, Elisabeth Nolan, Susan Shepler

**Affiliations:** 1 School of International Service, American University, Washington, District of Columbia, United States of America; 2 Center on Health, Risk and Society, American University, Washington, District of Columbia, United States of America; Armed Forces Health Surveillance Center, UNITED STATES

## Abstract

**Background:**

Future infectious disease epidemics are likely to disproportionately affect countries with weak health systems, exacerbating global vulnerability. To decrease the severity of epidemics in these settings, lessons can be drawn from the Ebola outbreak in West Africa. There is a dearth of literature on public perceptions of the public health response system that required citizens to report and treat Ebola cases. Epidemiological reports suggested that there were delays in diagnosis and treatment. The purpose of our study was to explore the barriers preventing Sierra Leoneans from trusting and using the Ebola response system during the height of the outbreak.

**Methods:**

Using an experienced ethnographer, we conducted 30 semi-structured in-depth interviews in public spaces in Ebola-affected areas. Participants were at least age 18, spoke Krio, and reported no contact in the recent 21 days with an Ebola-infected person. We used inductive coding and noted emergent themes.

**Findings:**

Most participants feared that calling the national hotline for someone they believed had Ebola would result in that person’s death. Many stated that if they developed a fever they would assume it was not Ebola and self-medicate. Some thought the chlorine sprayed by ambulance workers was toxic. Although most knew there was a laboratory test for Ebola, some erroneously assumed the ubiquitous thermometers were the test and most did not understand the need to re-test in the presence of Ebola symptoms.

**Conclusion:**

Fears and misperceptions, related to lack of trust in the response system, may have delayed care-seeking during the Ebola outbreak in Sierra Leone. Protocols for future outbreak responses should incorporate dynamic, qualitative research to understand and address people’s perceptions. Strategies that enhance trust in the response system, such as community mobilization, may be particularly effective.

## Author Summary

To decrease the severity of epidemics in countries with under-developed health system capacity to control outbreaks, lessons can be drawn from the Ebola outbreak in West Africa. This is the first study, to our knowledge, to use qualitative research methods to understand community members’ perceptions of using the Ebola response system during the outbreak in Sierra Leone. We conducted this study in two of the most populous districts during a time when there were still a high number of Ebola-related fatalities, and the Ebola response system had been scaled up. While national household surveys demonstrated high levels of intent to use the response system at the time, epidemiological reports suggested that there were delays in seeking testing and treatment. Our use of semi-structured in-depth interviews, as well as an ethnographer with experience in Sierra Leone, enhanced our ability to elicit people’s fears and misperceptions. Concerns about the response system clustered around three key themes: fears of calling the national hotline, negative perceptions of the chlorine spray, and misperceptions about the Ebola laboratory test and the need to re-test. These fears and misperceptions likely delayed people from seeking care. Our results lend support to the argument that trust in the public health response system was integral to citizens’ use of the system. We make several recommendations for how trust could have been enhanced during the Ebola outbreak. Protocols for future outbreaks should incorporate dynamic and qualitative research both to understand perceptions of the response system and to use these data to inform a more effective response.

## Introduction

Countries without the capacity to conduct surveillance, monitor, or control outbreaks are particularly at risk for negative consequences of infectious disease epidemics [1]. These countries’ inability to swiftly contain infectious diseases can result in global vulnerability to epidemic spread [2]. Since the Ebola outbreak in West Africa, there have been calls to improve the technical capacity for outbreak response [1,2]. Lessons from the Ebola outbreak can inform the development of future effective epidemic responses in under-resourced settings.

As of January 2016, Ebola resulted in 3,956 cumulative confirmed deaths in Sierra Leone [3]. One study estimated that Ebola likely killed more people in Sierra Leone in 2014 than the second (lower respiratory infections) and third (HIV/AIDS) leading causes of death and may have killed more people than the leading cause of death (malaria) [4]. A weak health system is partly to blame for Sierra Leone’s devastating experience of the 2014–16 Ebola virus disease outbreak (Ebola) [5–7].

In December 2014, the U.S. Centers for Disease Control (CDC) reported that Sierra Leone had more Ebola cases than Guinea and Liberia and that a significant number of Ebola-infected persons in Sierra Leone were being identified only after death, suggesting that these persons were either not captured by the surveillance system or were reluctant to use the response system [8]. Furthermore, WHO reported that between August and December 2014 the time between the onset of Ebola symptoms and hospitalization averaged two to three days [9]. This time lag may be explained by a reluctance to seek care. Nevertheless, in September 2014, over 90% of participants in a national household survey said they would go to a hospital or health facility if they thought they had Ebola [10]. In December 2014, 95% agreed that a person had a higher chance of survival if s/he immediately went to a health facility [11].

Although Sierra Leoneans reported high intentions for using the response system, previous anthropological studies of Ebola outbreaks elsewhere in Africa revealed apprehension about using it. These studies cited various factors including high Ebola-related fatality rates in hospitals, the isolation of infected patients, and the fear induced by the response workers’ full-body personal protective suits as reasons for people’s apprehension [12, 13]. Patients fled hospitals and refused to refer loved ones for treatment [13]. These studies identified a need for qualitative research on the barriers to trusting the public health response system during outbreaks [12].

Trust has received increased attention as a key concept determining personal compliance with and the overall success of public health efforts in developing countries [14–16]. Trust is conceptualized as a person or group’s perception of the health system, as well as their confidence in the system to competently deliver health services and contribute to overall social well-being [16,17]. Trust is influenced by individual and peer or family members’ experiences of the system, the reputation of the system, and messages from the media [15,16]. For example, a study from Tanzania demonstrated that pregnant women’s low trust, based in their experiences of poor quality services, impeded their use of rural maternal health care [14]. In a recent review article, Cairns et al. argued that risk communications moderate the relationship between trust and individual behavior during communicable disease outbreaks [15]. They suggested that designing credible and effective risk communication messages requires in-depth assessment of public understanding and risk perceptions at multiple time points during an epidemic [15].

The purpose of our study was to explore Sierra Leoneans’ perceptions of and intentions for using the Ebola response system at a critical time during the outbreak. We did not have any a priori hypotheses, but aimed to inform ongoing public health efforts to minimize Ebola’s spread. We used semi-structured in-depth interviews and our research team included an anthropologist with over twenty years’ experience conducting research in Sierra Leone (third author). Our inductive analysis revealed that trust was related to several of our findings.

## Methods

### Setting

This study occurred during January through March 2015 in Western Urban and Bo Districts, the two most populous districts in Sierra Leone. When formative research for the study commenced (1 January 2015), Sierra Leone had 248 new confirmed cases that week, the highest number in any of the three affected countries [18]. Western Urban, including Freetown, had the most new confirmed cases (93) [18]. Bo District, although heavily affected from September to November 2014 (with as many as 33 cases per week), reported only three new cases in the first week of 2015 and even fewer as the outbreak continued [18].

### The Response System

Our research occurred just after the rapid scale-up of the response system in Sierra Leone [6]. In January 2015, there were 4.6 beds for every confirmed and probable case, compared to 1.0 bed in October 2014 [19]. By January 2015, there were eleven laboratories nationwide, and every district had a laboratory and a contact tracing team to monitor quarantined individuals [19]. A national hotline (call 117), established on August 5, 2014, responded to reports of suspected Ebola cases and reports of any deaths, and also provided information about Ebola to citizens [20].

**Fig 1:**
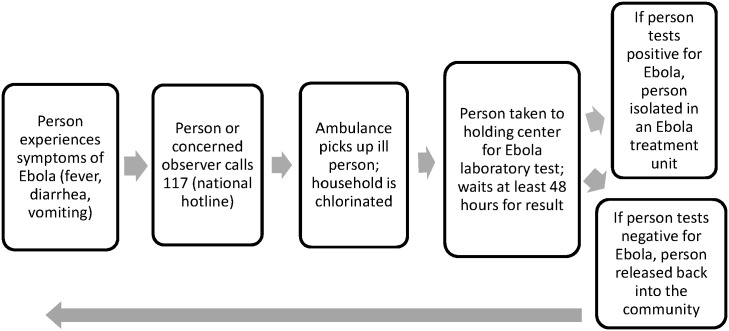
The Ebola response system in Sierra Leone, from the perspective of a potential user, as observed in January 2015.

Fig 1 illustrates the Ebola response system from the perspective of a potential user, as confirmed by our observations during fieldwork.

There were three ways to meet the “suspected Ebola case” definition: 1) a temperature of greater than 100.4°F (38.0°C) and three or more Ebola symptoms (vomiting, diarrhea, abdominal pain, headache, joint pain, fatigue, or unusual bleeding); 2) a fever and contact with a confirmed case in the preceding three weeks; or 3) unexplained bleeding [8]. Those who experienced any of these symptoms or noticed these symptoms in others were encouraged to call 117 [20]. The call center dispatchers alerted district-level response teams [20]. Ambulance workers dressed in personal protective equipment (PPE) arrived to transport suspected cases to holding centers. The ambulance workers also sprayed chlorine at homes of suspected cases in order to inactivate the virus [21]. Temporary holding centers isolated suspected Ebola patients awaiting laboratory test results [22]. Due to inefficiencies in the transportation and reporting systems, returning results on an Ebola test took a minimum of 48 hours [22]. People confirmed to have Ebola then moved to a free-standing Ebola Treatment Unit for isolation and care [22]. A contact tracing team then worked to find and monitor all those with whom the infected person had had contact [23]. Those confirmed not to have Ebola were released back into the community and told to return for testing if they developed Ebola symptoms.

### Ethics

We reviewed the consent form verbally with each potential participant and obtained verbal consent. Our ethics committees approved the use of verbal consent in part because the only record linking the participant to the research was the consent document. Furthermore, we anticipated that some participants may not have wanted their names recorded for fear of stigma and that some participants would be illiterate. Verbal consent was requested and then documented by the study interviewer. To preserve anonymity, we conducted all interviews in a semi-private public space where the content of the interview could not be overheard, we did not collect names, and we redacted identifying information from the transcripts. As a token of appreciation, we provided each participant with a bottle of hand sanitizer, a valued but not readily available commodity. The Institutional Review Boards (IRB) at American University (AU) and the Office of the Sierra Leone Ethics and Scientific Review Committee approved our ethical procedures. Unfortunately, we did not obtain permission from participants to release their data for public use, and thus the AU IRB did not grant permission to release the data.

### Interviews

The eligibility for our study included: being aged 18 years and older; having had no known contact with an Ebola-infected person for the past 21 days (to protect ourselves and our future contacts); and willingness to provide informed consent. We conducted semi-structured in-depth interviews with a convenience sample of thirty Sierra Leoneans split evenly between Western Urban and Bo Town. We recruited participants by approaching people in public places such as marketplaces, roadsides, checkpoints, and previously quarantined settlements. Although congregating was prohibited at this time of the outbreak, there were still people publically socializing. We elected to recruit participants in public places rather than through non-governmental organizations in order to capture a more general population. We purposively interviewed participants who looked to be diverse along three demographic characteristics: gender, age and occupation. These characteristics were observed by the interviewer and then a potential participant was approached. No person refused to participate.

The interviews lasted thirty to forty minutes and were conducted in Krio by an experienced ethnographer (the third author) and a trained, local qualitative interviewer. Questions included people’s beliefs about Ebola, their perception of their risk for Ebola and what they had done to protect themselves. We asked participants to tell us what they would do if they or someone they knew developed a fever, and if they would call or had called the national hotline. We also asked about their perceptions of the Ebola laboratory test. Demographic characteristics captured included the participant’s age, occupation and sex.

### Data Analysis

All interviews were translated to English from Krio by two research assistants and the translations were verified by the third author. All authors read all English transcripts. Using Atlas.ti version 7, the second author assigned codes deductively from the interview guide and then inductively identified emergent codes from the data. The codes and quotes were reviewed by the lead investigator. Once the data were categorized, comparisons were made between participants. Data saturation was reached after several rounds of analysis by all authors. The most salient and common themes regarding respondent perceptions of the Ebola response system were described and matrices were used to summarize diverse perceptions related to each theme.

## Results

### Participant Demographics

We interviewed sixteen men and fourteen women aged 18–56. In approximately equal proportions, the categories of their occupations included: 1) business (trader, seamstress, manager); 2) social service (nurse, teacher, burial team driver); 3) petty business (car wash, gambling, roadside seller); 4) youth (students, young mother); and 5) community leaders (section chief, pastor, community-based organization leaders).

Nearly all participants reported that at the epidemic’s beginning they did not believe Ebola was real. Some believed Ebola was another known illness like cholera. Other explanations included government collusion:

“*Some people initially felt [Ebola] was politicized and that since the census was about to start*, *they felt [Ebola] was a way of reducing the population before the census*.”

—*Receptionist*

However, nearly all participants reported that they came to believe Ebola was real because they observed or knew of people dying, or because they heard that health professionals died:

“*Because I have seen two*, *three people that the ambulance has taken away*. *And the way I’ve seen them in the ambulance proves to me that Ebola is a reality and not a made-up story*.”

—*Trader*

**Fig 2:**
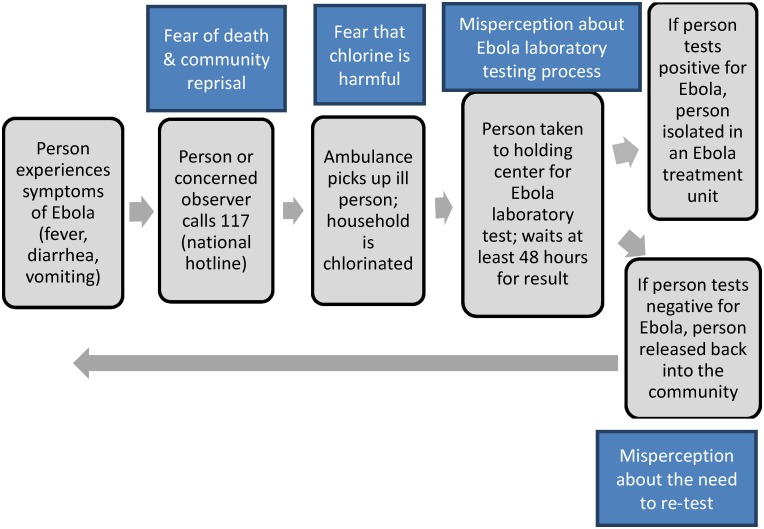
Fears and misperceptions of the Ebola response system in Sierra Leone, reported by research participants in January 2015.

Given that our participants believed in Ebola we asked them about their likelihood of using the response system. Our results are described below and summarized by Fig 2.

### Calling 117

In response to the question “what would you do if you had a fever?” very few participants said they would call 117 right away. A few participants said they would call, but only when the fever was very bad: if they could not walk, the fever persisted more than a few days, or it did not respond to other treatment. In about half of interviews, respondents said they would start by treating themselves with medicines normally used for other illnesses such as malaria, typhoid or cholera. Several participants claimed to know what the fever would feel like if it was Ebola, and that they would wait for those symptoms before reporting:

*Because based on what the medical people say, if it is high fever, then you will say this has gone beyond limit. Because there are times when we get malaria and common cold before this Ebola*. *So you cannot just start feeling feverish and you say you have to call 117*.

—*Business sector worker*

Three-quarters of participants said that if they got a fever they would go to a local health center before calling 117. One participant said that he could not afford to go to a health center and so he would pray. Another respondent said she would go to a health center, but she was afraid of being sent to an Ebola treatment unit.

Participants were more willing to call 117 if they observed symptoms in another person. However, about one-third of participants stated they were afraid of community members’ anger if they were to call. One person said that he would not call personally, but he was confident someone else in the community would. Some expressed fears that by calling they would never see the person again:

“*With 117*, *the ambulance takes people away and sometimes they never come back … I think this is what frightens people*.*”*

—*Community leader*

Only three participants had ever called 117. One person reported that he called 117 for his neighbor and the neighbor became very angry with him. Another person reported calling six times, not receiving a response, and then seeking other health care resources for the suspected case.

### Chlorine Spray

About one-third of participants expressed the belief that chlorine was harmful to people. Most believed danger arose when patients were put inside ambulances with chlorine fumes:

*Chlorine suffocates patients. If a patient has been vomiting and toileting then in the end he or she is suffocated with chlorine, because when they spray the patient in the vehicle they usually close the door where the patient will be placed and no air enters the cabin*.

—*Community leader*

However, some respondents who believed chlorine was harmful also used chlorine as a part of their preventative hand-washing to avoid spread of the disease. Some did not think that the chlorine in the hand-washing bucket was the same as that used by health workers to spray.

Two respondents even believed it might be the chlorine spray that was killing people and not Ebola:

*One day the team came to pick up a female patient … She was strong enough to walk from the house to the ambulance but when they arrived at the treatment center the lady became so weak and couldn’t walk because of the chlorine sprayed in the ambulance. It was the chlorine. She didn’t have Ebola. The chlorine weakened her. The effect of chlorine is just like when one is locked up in a newly painted room. It suffocates people just like Shelltox (insecticide spray)*.

—*Petty business worker*

### Ebola Laboratory Test

When asked what they knew about the laboratory Ebola test, nearly half of participants said they did not know anything. When asked explicitly about what they knew about the test, five respondents were only able to discuss the use of infrared thermometers as a pre-test for Ebola:

*I have heard about [the test] but I do not really know what it entails. There is a machine that I know they point at people to take their temperature. And the temperature should be at certain limit otherwise they will do further tests on you to prove whether the rise in temperature is not due to Ebola*.

—*Social services worker*

Four respondents mentioned the test occurring only after a person had died. Most did not have a clear idea of how the testing process worked. Many respondents expressed concern or even disbelief that the testing process was valid:

*I hear they take the blood specimen of people but even there it is not clear to me. They can take the blood of somebody under the pretext that he/she has Ebola but at the end of the day you will hear that that person has been discharged*.

—*Business sector worker*

Some also misunderstood the need to re-test for Ebola. Several participants discussed that they did not understand how a person who tested negative for Ebola could have subsequently died because their original Ebola “*status was negative*.”

## Discussion

While national surveys showed high levels of intent to use the Ebola response system during the 2014–2015 outbreak in Sierra Leone [10], our qualitative findings reveal apprehension that may have contributed to the low usage rates. Several of our findings, including participants’ reluctance to call the national hotline and endure the chlorine sprayed by ambulance workers, point to a lack of trust in the Ebola response system. While other researchers have reported on people’s fears of the response system’s management of burials and quarantine during the West African outbreak [24–26], we explore aspects of resistance that, to our knowledge, have not yet been discussed. Below we reflect on our findings, discuss how the response system might have enhanced trust, and provide concrete suggestions for the public health response during an Ebola outbreak.

Participants in our study reported reluctance to call the national hotline if they had a fever because they were afraid they would not return alive. Participants reported seeing many deaths at the beginning of the epidemic which provided the rationale for these fears, as did the high Ebola-related fatality rate in 2014 in Sierra Leone [22,27]. Although Ebola treatment and surveillance capacity had increased greatly by the time of our research [6], participants’ early impressions likely remained salient in their attitudes towards accessing the response system. Trust in a public health system is enhanced when the system demonstrates accountability to its users [14]. Thus, one implication of this finding is that demonstrating an effective response very early during an outbreak is useful for gaining citizens’ trust and ongoing use of a response system.

Participants also reported they would ensure that a fever was due to Ebola, rather than another illness, before seeking care. In the interim they would try to self-medicate or visit local trusted health care facilities. This finding is a potential explanation for why there was a two to three day delay between onset of Ebola symptoms and hospitalization [9]. However, it is important to note that local facilities often did not have adequately trained personnel to deal with Ebola [6]. Therefore, we suggest that public health efforts directly address the similarities between Ebola and other illnesses, as well as encourage citizens to use specialized health facilities.

Some participants feared that the chlorine used by ambulance workers was toxic and potentially lethal. Participants’ fears may have resulted from real observations. There was a reported case of a nurse experiencing respiratory distress and brief loss of consciousness after inhalation of highly concentrated chlorine gas at an Ebola treatment unit in Sierra Leone [28]. To our knowledge, public health messaging did not substantially address the fear of chlorine until May 2015 [29]. Our participants were not fearful of the chlorine they used to wash their hands, referred to as “Britex” in Sierra Leone. Thus, we suggest that public health messaging use local terms to describe the chlorine spray and reassure citizens that it is not dangerous.

We found that participants did not understand the need to re-test for Ebola should symptoms develop. Several participants used the word “status” to refer to the Ebola test. It is possible that they were appropriating HIV prevention language which encourages citizens to “know your HIV status.” Thus, people diagnosed as Ebola-negative may have returned to communities thinking they were Ebola-free, despite a high likelihood of exposure while waiting at holding centers. Although the importance of re-testing may be challenging to communicate, it is crucial to identify the most relevant information to distribute.

Moreover, public health messages persisted in communicating the “Ebola is real” message, although most of our participants already believed that. Building trust in a response system requires institutions to distribute risk reduction messages that are tailored to the public’s perceptions over time [15]. Our findings suggest that as an outbreak persists, more complex messages should be distributed to the public like the need for repeat testing and the effects of the chlorine sprayed by ambulance workers.

Sierra Leoneans’ distrust of the state and lived experiences of corruption may have also negatively affected perceptions of the government-run Ebola response system [6,30,31]. The devastating civil war created a breakdown of civic trust, whose rehabilitation is hampered by continued poor governance and corruption, including in the health care system [30]. Mistrust in a state’s ability to respond to an epidemic is not unique to Sierra Leone. A quantitative study of Italian citizens during January to March 2015 found that they did not trust in their institutions’ preparedness for Ebola [32]. Health care systems can build trust by exhibiting technical competence, transparency, and reliability during outbreaks and developing strategic response plans during non-outbreak periods [14,15,17].

Using cultural insiders and leaders to address people’s misperceptions and demonstrate accountability to the public can also enhance trust and encourage health system use [26,33]. A case study of community resistance from February 2015 in a village of the Guinean Forest region provides an example [34]. To transform the villagers’ perception that the Ebola treatment centre (ETC) sold people’s organs for trade, the organization responsible for the ETC asked four survivors to publicly discuss their positive experiences in the ETC. After the survivors’ testimonials, the villagers cooperated with the contact tracing and surveillance team. An understanding of community perceptions and the use of cultural insiders were successful strategies in this case.

There are several limitations of our study. We interviewed people during a time when resources were adequate for the cases reported. Following the recommendations by Cairns et al., we ideally would have interviewed people at multiple time points during the epidemic to assess their dynamic perceptions of the response system [15]. In addition, although our sample was diverse, we cannot claim it was representative of a larger population. Restrictions on movement during the Ebola outbreak constrained our interviews to two urban parts of Sierra Leone, preventing a broader reach.

In conclusion, our research during the Ebola outbreak revealed that Sierra Leoneans had several fears and misperceptions about using the response system that have heretofore not been reported. We used in-depth interview methods and an experienced ethnographer, allowing us to explore lower-than-ideal usage of the response system in the face of national surveys that demonstrated uniformly positive attitudes towards it. Our findings and reflections on trust as a conceptual framework contribute several concrete suggestions for public health response during an Ebola outbreak. These suggestions include the need to conduct multiple and in-depth assessments on the public’s perceptions of the response system and to use these data to distribute more complex public health messages as the epidemic progresses.

## Supporting Information

S1 Checklist STROBE Checklist (DOC)
